# Scoping review: (Bio)markers for the prognostication of breast cancer recurrence

**DOI:** 10.1186/s12885-025-14515-z

**Published:** 2025-07-01

**Authors:** Rigon Sallauka, Matej Horvat, Maja Ravnik, Hatem Rashwan, Umut Arioz, Izidor Mlakar

**Affiliations:** 1 HUMADEX Group, Faculty of Electrical Engineering and Computer Science, University of Maribor, Maribor, Slovenia; 2 Oncology Department, University Medical Center Maribor, Maribor, Slovenia; 3 Department of Computer Engineering and Mathematics (DEIM), Universitat Rovira I Virgili, Tarragona, Spain

**Keywords:** Breast cancer recurrence, Minimally invasive markers, Scoping review, Radiological markers, Clinical markers, Histopathological markers, Prognostic

## Abstract

**Background:**

The aim of this study is to gain a comprehensive understanding of the latest advancements in breast cancer recurrence markers, with the aim of identifying minimally invasive or minimally intrusive markers as necessary approach for screening for breast cancer recurrence.

**Methods:**

We followed PRISMA guidelines, systematically searching Web of Science, Scopus, and PubMed from 2010 to December 2023 for secondary papers on breast cancer markers of recurrence. Keywords used to search the databases include but are not limited to: “breast cancer recurrence”, “markers”, “radiology”, “pathology”, “clinical features”. Studies focusing solely on outcomes after recurrence, such as survival or treatment response, were excluded to ensure the review targeted markers relevant to early prediction. The search was limited to English language. Selected papers underwent screening process according to inclusion/exclusion criteria, and data extraction included publication details, markers, marker modality, among others.

**Results:**

The number of papers considered for this review was 1,138. After two phases of screening process, a total number of 28 reviews were included in this scoping review. We have categorized markers into radiological, clinical, and histopathological types. Among the most relevant clinical markers correlated with breast cancer (BC) recurrence are clinical stage, carcinoembryogenic antigen (CEA), and cancer antigen 15.3 (CA 15.3). We have also identified that the following radiological markers are the most mentioned markers associated with recurrence: mammographic density (MD), tumor heterogeneity, most enhancing tumor volume (METV), radiomic features, and more. Furthermore, we identified nuclear grade, microenvironment heterogeneity, estrogen receptor (ER), androgen receptor (AR), human epidermal growth factor receptor 2 (HER2), Ki-67 antigen, as the most significant histopathological markers of breast cancer recurrence.

**Conclusion:**

This review identified promising markers for breast cancer recurrence in three categories: clinical, radiological and histopathological. General practitioners can leverage these insights for enhanced pre-screening, aiding in earlier detection and intervention, thus improving patient outcomes. Unclear cut-off values and disagreement on their use remain obstacles.

## Introduction

Breast cancer is the most frequent cancer diagnosed in women worldwide. Breast cancer remains a significant global health challenge, with over 2.3 million new cases diagnosed annually [1]. Despite significant advances in treatment, recurrence remains a prevalent issue, accounting for approximately 40% of breast cancer deaths [2]. Accurately predicting the likelihood of recurrence is essential for better risk assessment, informed treatment choices, and personalized patient care.

Markers are essential in the management of patients with breast cancer, particularly in guiding the choice of systemic therapy. The biological indicators reflect a patient's individual disease characteristics and response to treatment, enabling personalized treatment plans that maximize efficacy and minimize adverse effects. Specifically, they can provide valuable insights into a patient's tumor biology, including its molecular makeup and genetic mutations. This information is crucial for determining the most effective therapies, such as targeted therapies or endocrine therapy, which are specifically designed to target the underlying molecular drivers of the cancer. Therefore, prognostic markers have the potential to improve patient outcomes, that leads to longer survival rates, reduced recurrence rates, and improved quality of life. Their use in breast cancer management is a significant step forward in personalized medicine, customizing treatment to the characteristics of each patient. Clinical markers and symptoms that are non-invasive or minimally intrusive can aid in the prognosis of breast cancer recurrence by offering valuable insights into the patient's status without the need for invasive interventions. Utilizing these markers and symptoms enables ongoing monitoring of patients and the identification of any changes that could signal a recurrence.

However, since the field of markers for breast cancer recurrence is vast and diverse, it is unclear what kind of information is available in literature. Given the fact that we encountered a vast number of reviews done in this topic, a scoping review of the reviews was conducted to map the research done in the area and identify any existing gaps.

The aim of this scoping review is to explore the most known factors that are correlated with breast cancer recurrence, with a focus on clinical markers, followed by an investigation into more intrusive markers like radiological and histopathological, to identify recurrence predictors. To align with this aim, we focused on studies that examined predictors of recurrence prior to its occurrence, rather than those addressing outcomes following recurrence, such as survival or treatment response. This insight gives us the opportunity to monitor patients over time using non- or minimally intrusive markers and detect changes indicating recurrence. To the best of our knowledge, this is the first scoping review to synthesize existing review articles specifically focused on prognostic markers for breast cancer recurrence.

## Methods

We followed the guidelines defined by the Preferred Reporting Items for Systematic Reviews and Meta-Analyses extension for Scoping Reviews (PRISMA-ScR) [3].

### Search strategy

For this review, we did a systematic search in the following platforms: Web of Science (https://www.webofscience.com/), Scopus (https://www.scopus.com/), and PubMed (https://pubmed.ncbi.nlm.nih.gov/). We retrieved all papers in the English language, in the time span of 2010–2023. The search also included a “Review” flag, that selected all papers marked as review papers from the platforms.

We used a combination of the following terms to search titles, keywords, and abstracts of papers: “breast cancer recurrence”, “medical imaging”, “image analysis”, “image fusion”, “texture analysis”, “symptoms”, “risk”, “markers”, “markers”, “clinical features”, “serum markers”, “clinicopathological”, “radiology”, “pathology”, “digital breast pathology”, “blood test”, “radiomics”, “histology”, “MRI”, “CT”, “PET”, “mammography”, “dbt”. A sample of the original search string used to search in Scopus platform is provided here:

TITLE-ABS-KEY(breast AND cancer AND recurrence) AND TITLE-ABS-KEY ((medical AND imaging) OR image OR (image AND analysis) OR (image AND fusion) OR (texture AND analysis) OR imaging) AND TITLE-ABS-KEY(symptoms OR risk OR markers OR marker OR (clinical AND features) OR clinicopathological OR (serum AND markers)) AND TITLE-ABS-KEY (radiology OR pathology OR (digital AND breast AND pathology) OR (blood AND test) OR radiomics OR histology OR mri OR ct OR pet OR mammography OR dbt) AND DOCTYPE (re) AND LANGUAGE(nglish) AND PUBYEAR > 2009 AND PUBYEAR < 2024.

### Eligibility criteria

We used the following criteria to screen the papers.

### Inclusion criteria

1. Paper type: secondary papers (systematic reviews, scoping reviews, and narrative reviews).
2. Papers talking about breast cancer markers.
3. Papers talking about prognosis or prediction of recurrence of breast cancer.
4. Papers talking about radiological, histopathological, or clinical markers.
5. Papers in English language.
6. Papers published between 2010–2023.

### Exclusion criteria

1. Original papers – primary papers.
2. Papers not on topic of breast cancer markers.
3. Papers not on topic of breast cancer recurrence prognosis or prediction (diagnosis or detection)
4. Papers not talking about radiological, or histopathological, or clinical markers (genetics)
5. Papers not in English language.
6. Papers not published between 2010–2023.

We focused on studies that examined predictors of breast cancer recurrence prior to its occurrence, consistent with our aim to identify markers that may help monitor patients over time and detect early signs of recurrence. Studies where “prognosis” referred exclusively to outcomes following recurrence, such as survival or treatment response, were excluded during screening. This distinction ensured that the selected literature reflected our focus on anticipating recurrence, rather than managing it after it has already occurred.

### Study selection

After removal of duplicates, papers underwent two screening phases. In the first screening phase, titles, keywords, and abstracts of papers were screened by six independent reviewers against inclusion and exclusion criteria. The full text of the included papers from the first phase was screened by the same five reviewers, and those that did not meet the inclusion criteria were excluded. Any disagreements between reviewers at any stage of the process were resolved through discussions.

### Data extraction

A data charting table was created for the data collection process. Data extracted from each study include in whole or a combination of year of publication, type of review, markers, their modality, and findings. Two reviewers independently extracted data from each included review to ensure consistency and accuracy. Discrepancies between reviewers were resolved through discussion or consultation with a third reviewer. The charting table was iteratively refined during the data extraction process to accommodate new information or emerging themes.

### Data synthesis

We did a descriptive and thematic synthesis approach, appropriate for scoping reviews. After charting the extracted data, we categorized the identified markers based on their modality: clinical, radiological, or histopathological. Within each category, we grouped specific markers that recurred across multiple studies and noted the frequency and context of their appearance. Common trends, recurring themes, and notable gaps in the literature were highlighted narratively to provide a structured overview of the current state of evidence on breast cancer recurrence markers.

## Results

**Fig. 1:**
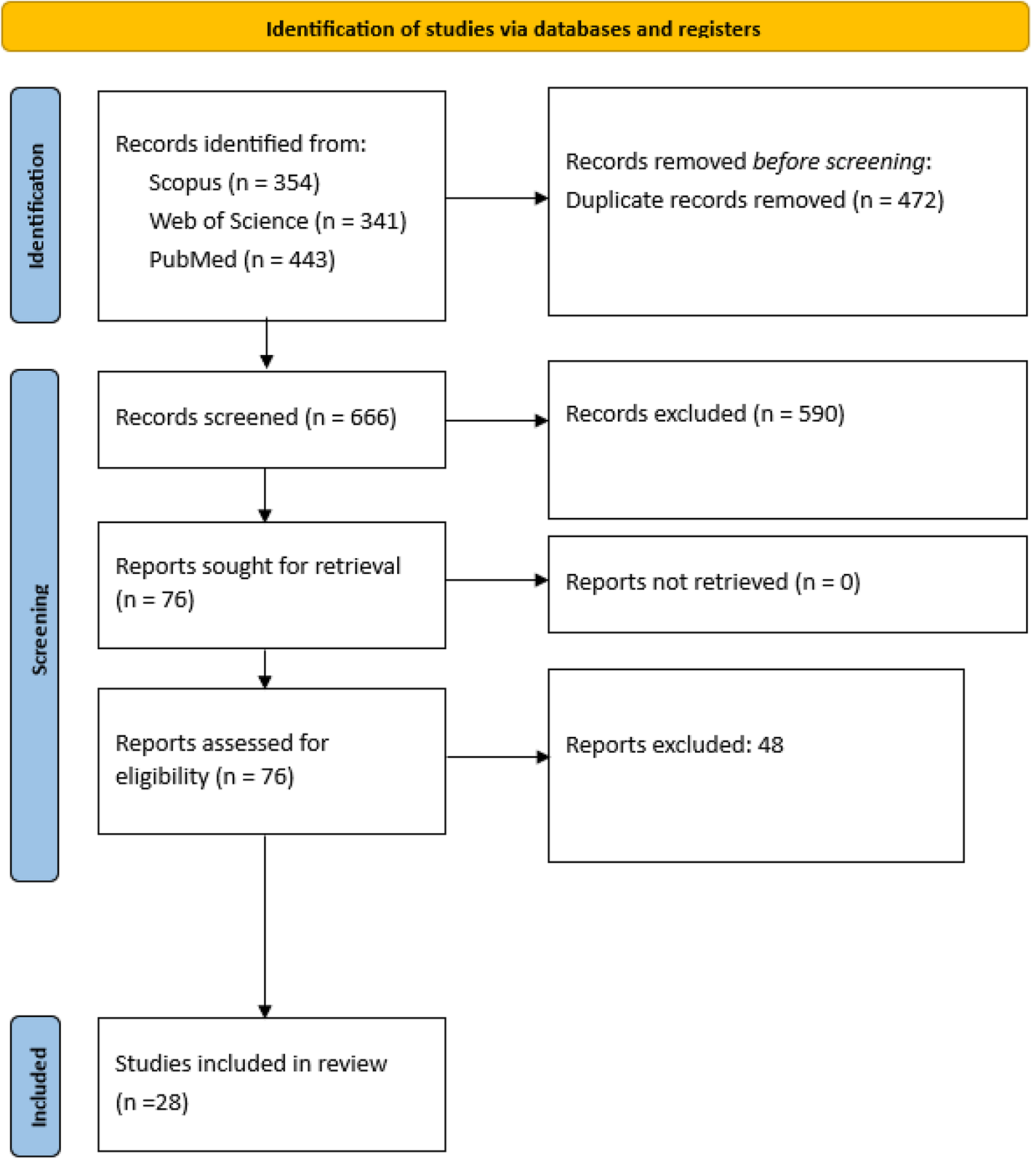
PRISMA-ScR flowchart

We retrieved 1,138 papers from the three platforms mentioned above. The number of duplicates was 472, so the number of papers that underwent a screening process was 666. After the removal of duplicates a first screening of articles was done by five reviewers (RS, MH, MR, HR, UA, IM), where the titles, abstracts, and keywords of papers were screened based on the inclusion and exclusion criteria mentioned above. The second process of screening was conducted on the 76 papers that were included in the first screening process, where full text of papers was screened against inclusion and exclusion criteria. We ended up with the final list of 28 papers. Figure 1 presents the PRISMA flowchart for the screening of papers.

Table 1 gives a summary of the findings that we are going to discuss here.

**Table 1:** Summary of the identified markers of breast cancer recurrence

*Maker*	*Marker category*	*Number of papers*	*List of papers*
*CA 15.3*	clinical	5	Djassemi N. et al., Evangelista, L. et al., Groheux, D. et al., Kruse, V. et al., Paydary, K. et al
*CEA*	clinical	4	Djassemi N. et al., Evangelista, L. et al., Groheux, D. et al., Paydary, K. et al
*MD*	radiological	4	Kanbayti I.H. et al., Shawky M.S. et al., Huo, C.W. et al
*clinical stage*	clinical	3	Bazan J.G. et al., Ming, Y. et al., Trimboli, R. M. et al
*Oncotype DX recurrence score*	clinical	3	Fitzgerald, J. et al., Solin L.J. et al., Krug, D. et al
*METV*	radiological	3	Conti, A. et al., Reig, B. et al., Crivelli, P. et al
*SUV*	radiological	3	Lovinfosse P. et al., Ming, Y et al., Paydary, K. et al
*MTV*	radiological	3	Lovinfosse P. et al., Ming, Y. et al., Urso L. et al
*HIZE, HISZE*	radiological	3	Lovinfosse P. et al., Sollini M. et al., Urso L. et al
*HER2*	histopathological	3	Hanna W.M. et al., Trimboli, R. M. et al., Krug, D. et al
*age*	clinical	2	Solin L.J. et al., Trimboli, R. M. et al
*BPE*	radiological	2	Liao, GJ. et al., Rella, R. et al
*Ki-67*	histopathological	2	Hanna W.M. et al., Krug, D. et al
*nuclear grade*	histopathological	2	Hanna W.M. et al., Hayward, M.K. et al
*tumor size*	clinical	1	Solin L.J. et al
*number of involved ALNs*	clinical	1	Bazan J.G. et al
*TLG*	radiological	1	Ming, Y. et al
*ADC*	radiological	1	Yao F.-F. et al
*tumor heterogeneity*	radiological	1	Chitalia R.D. et al
*Wavelet features;*	radiological	1	Chitalia R.D. et al
*ER expression*	histopathological	1	Hanna W.M. et al
*TILs*	histopathological	1	Trimboli, R. M. et al
*microenvironment heterogeneity*	histopathological	1	Fitzgerald, J. et al
*morphometric information from H&E samples*	histopathological	1	Fitzgerald, J. et al
*spatial heterogeneity*	histopathological	1	Fitzgerald, J. et al
*tumor size*	histopathological	1	Hanna W.M. et al
*high grade*	histopathological	1	Hanna W.M. et al
*Comedonecrosis*	histopathological	1	Hanna W.M. et al
*Histologic growth pattern*	histopathological	1	Hanna W.M. et al
*multifocal DCIS*	histopathological	1	Hanna W.M. et al
*AR*	histopathological	1	Hanna W.M. et al

Abbreviations: *CA* Cancer antigen, *CEA* Carcinoembryogenic antigen, *MD* Mammographic density, *METV* Most enhancing tumor volume, *SUV* Standardized uptake value, *MTV* Metabolic tumor volume, *HER2* Human Epidermal Growth Factor Receptor 2, *HIZE* High-intensity zone emphasis, *HISZE* High-intensity short-zone emphasis, *ALN* Axillary lymph node, *TLG* Total lesion glycolysis, *ADC* Apparent diffusion coefficient, *ER* Estrogen receptor, *TIL* Tumor infiltrating lymphocites, *H&E* Hematoxylin and eosin, *DCIS* Ductal carcinoma in situ, *AR* Androgen receptor, *BPE* Background parenchymal enhancement

### Clinical markers

Clinical markers are measurable indicators that reflect normal or pathological biological processes, pharmacological responses, or therapeutic interventions. They can include a wide range of substances, molecules, or physical characteristics found in blood, urine, or tissues. Clinical markers play a crucial role in medicine by providing objective information about a patient's health status, disease progression, response to treatment, and overall prognosis.

We have identified several clinical markers across different papers that were associated with recurrence of breast cancer. Two serum markers, namely cancer antigen 15.3 (CA 15.3) and carcinoembryogenic antigen (CEA) are the most relevant markers correlated with recurrence. Five papers associated CA 15.3 with BC recurrence, while four papers found that CEA is also a relevant marker predicting BC recurrence. Djassemi N. et al. reported that elevated serum tumor markers can be useful prognostic markers of recurrence in asymptomatic breast cancer patients [4]. In another review, high levels of CA 15.3 marker indicate, almost certainly, the presence of metastatic disease; however, low CA 15.3 levels do not exclude the presence of malignancy [5]. Three other reviews, reported that an increase in levels of serum tumor markers in asymptomatic patients suggest recurrence [6–8]. Clinical stage is another factor that is correlated with recurrence identified across a number of reviews. Bazan J.G. et al. reported that clinical stage IIIB disease or greater, ≥ 4 involved ALNs were identified as predictive of loco-regional recurrence (LRR) [9]. Trimboli et al. also found that stage remained the strongest predictor of the risk of recurrence, along with age, while Ming, Y. et al. showed that TNM staging parameters were significantly different between patients with and without recurrence [10, 11]. Oncotype DX score, a genomic test used in breast cancer patients to help predict the risk of cancer recurrence, is also mentioned in three reviews as a factor in predicting BC recurrence [12–14]. While genomic tests such as Oncotype DX are based on gene expression analysis and thus fall under molecular diagnostics, we included it in the category of clinical markers due to its widespread use as a validated clinical decision-making tool in breast cancer management.

### Radiological markers

Radiological markers, which are also known as imaging markers, are anatomic, physiologic, biochemical, or molecular parameters detectable with imaging methods, such as mammography, CT scans, MRIs, etc., that are used to establish the presence or severity of disease [15]. These markers can provide valuable information about a patient's health, including the presence and severity of disease, the response to treatment, and the risk of future complications.

**Fig. 2:**
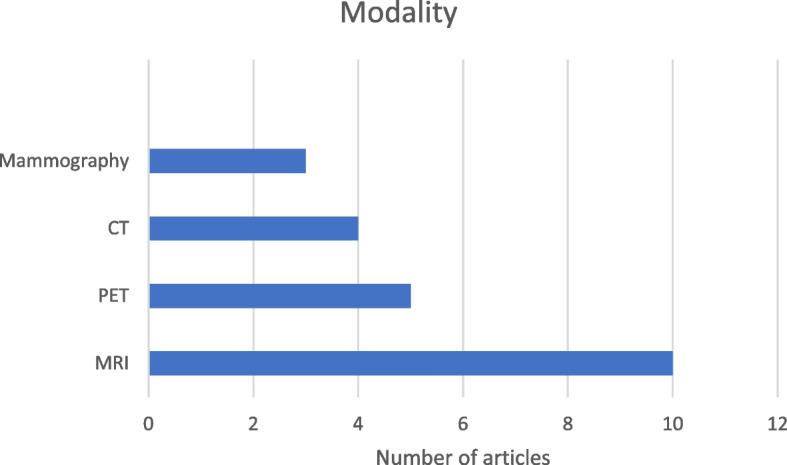
Most used imaging techniques for marker extraction. x-axis represents the number of articles mentioning markers extracted by the radiological modality. y-axis represents the modality

Our review found radiological markers mentioned in 16 of the analyzed papers. Magnetic Resonance Imaging (MRI) was the most common imaging technique used to extract these markers, followed by Positron Emission Tomography (PET), Computed Tomography (CT), Mammography, and Ultrasound (US). As shown in Fig. 2, MRI appeared in 10 papers, PET in 5, CT in 4, and Mammography in 3.

Radiomics is a rapidly evolving field of research concerned with the extraction of quantitative metrics-the so-called radiomic features within medical images. Radiomic features capture tissue and lesion characteristics such as heterogeneity and shape and may, alone or in combination with demographic, histologic, genomic, or proteomic data, be used for clinical problem solving [16]. We have found that there are several radiomics features associated with breast cancer recurrence mentioned in literature. Chitalia R.D. et al. found that wavelet texture features from within tumor subregions partitioned by pharmacokinetic behavior and concluded that the spatial frequency texture pattern captured using wavelets within the heterogeneous subregions could serve as a strong prognostic marker for predicting risk of tumor recurrence. The same paper mentions tumor heterogeneity as potential independent prognostic marker [17]. Ming et al. reported that high-intensity zone emphasis (HIZE) from the intensity size-zone matrix was significantly correlated with recurrence [10]. HIZE (high-intensity zone emphasis) and HIZSE (high-intensity short-zone emphasis) were also reported to be significant predictors of recurrence in two other reviews [18, 19].

Apart from radiomic features, other markers were reported to be correlated with risk of recurrence. Mammographic density (MD) is mentioned in several papers as a prognostic marker of breast cancer recurrence. Baseline mammographic density (BMD) is associated with recurrence, whereas mammographic density reduction (MDR) is associated with a reduced risk of recurrence [20]. In two other papers higher level of mammographic density is mentioned to be associated with an increased risk of local recurrence or a second primary BC [21, 22]. Another marker associated with recurrence is most enhancing tumor volume (METV). METV is generally predictive of future recurrence with a high value indicative of a future recurrence and a low value indicative of the absence of recurrence [23–25]. Liao et al. and Rella et al. reported background parenchymal enhancement (BPE) as a prognostic marker of recurrence. They found that a strong positive association exists between an increase in BPE and risk of recurrence [26, 27]. Lovinfosse et al. reported that an unsupervised automatic clustering algorithm defined 3 iconographic patterns based on standardized uptake value (SUV), metabolic tumor volume (MTV), total lesion glycolysis (TLG) and heterogeneity features, which predicted tumor recurrence [28]. SUV and MTV were also mentioned as being correlated with risk of recurrence in several other papers [6, 10, 19]. Yao et al. showed that the apparent diffusion coefficient(ADC) difference value (between the 5th and 95th percentiles) was significantly lower in the low-risk than in the intermediate or high-risk recurrence groups [29].

### Histopathological markers

Histopathological markers are molecular or cellular characteristics observed in tissue samples, that are used to diagnose a disease, assess its severity, or predict its course.

Fitzgerald, J, et al., in a short review explores how integrating AI can improve breast cancer detection, measurement, categorization, outcome prediction, and leverage machine learning to analyze markers. Microenvironment heterogeneity, together with key genomic alterations, was found to be useful to identify patients at high risk of relapse. Other markers reported in this review include morphometric information from H&E samples (mitosis, architectural patterns, nuclei), and spatial heterogeneity of immune infiltration [12]. Hanna W.M. et al. reported several markers associated with recurrence of a type of breast cancer, DCIS (ductal carcinoma in situ). Morphological features of DCIS that have been most consistently found to predict the risk of local recurrence in retrospective and prospective studies include high nuclear grade, presence of comedonecrosis, larger lesion size, and multifocal DCIS. Also other factors were found to be valuable in prognosis of cancer recurrence such as: histologic growth pattern, ER expression, AR expression (its ratio with ER), Ki-67, and HER2 [30]. Nuclear grade was identified as a prognostic marker also in another review, along with tumor infiltrating lymphocytes (TIL) density [31]. Human Epidermal Growth Factor Receptor 2 (HER2), a protein found on the surface of breast cancer cells, is reported to be a prognostic factor of recurrence in two other reviews, apart from the previously mentioned review [11, 14]. In the review by Khosravi-Shahi et al., surgical margins < 1 cm are reported to be associated with the highest risk of local recurrence. Other factors correlated with recurrence include mitotic activity, and tumor necrosis, while stromal cellularity, overgrowth, and atypia, along with heterologous stromal elements were significantly correlated with metastases [32].

### Synthesis of results

In summary, this scoping review identified a range of markers associated with breast cancer recurrence across three main modalities: clinical (e.g., CA 15.3, CEA, clinical stage), radiological (e.g., MD, METV, radiomic features), and histopathological (e.g., HER2, Ki-67, nuclear grade). CA 15.3 and mammographic density were the most frequently reported serum and imaging-based markers, respectively. This synthesis maps the current evidence landscape and supports the identification of minimally or non-intrusive markers that could aid in prognostication of recurrence.

## Discussion

In this scoping review we identified 23 secondary studies addressing markers of breast cancer recurrence. This review provides a comprehensive analysis of breast cancer recurrence markers across radiological, clinical, and histopathological categories. Radiological markers, particularly MRI-derived radiomic features, demonstrate promise in predicting recurrence risk. Clinical markers such as CA 15.3 and CEA, alongside established factors like clinical stage and Oncotype DX score, emerge as potential indicators. Histopathological analysis underscores the significance of microenvironment heterogeneity and established markers like nuclear grade, HER2 status, and immune cell infiltration in assessing recurrence risk. However, challenges persist, including unclear cut-off values for many markers and ongoing disagreements among clinicians regarding usability and relevance. Addressing these challenges will require integrating findings with advanced techniques like AI-powered analysis, offering significant potential for personalized risk stratification and improved breast cancer management. Moreover, the integration of non-invasive clinical markers and symptoms can enhance prognostication, empowering general practitioners to identify individuals at higher risk of recurrence and intervene proactively.

## Strengths & limitations

One of the key strengths of this review lies in its comprehensive and systematic search strategy, which spanned three major databases and adhered to PRISMA guidelines. This approach enabled a broad overview of the current landscape of breast cancer recurrence markers. However, there are also some limitations to consider. Many papers did not provide data in certain categories, and there is a notable absence of standardized reporting methodologies. Only English papers and papers in a 13-year span were used. Additionally, it's important to acknowledge the scarcity of available data in the topic, which could have impacted the comprehensiveness of our analysis. It is important to note that our search strategy did not specifically target genetic or molecular biomarkers, which may explain their absence from the included reviews. Oncotype DX, although technically a genomic assay, was included in this review because it appeared in several reviews and is widely used as a clinical decision-making tool in the management of breast cancer. Furthermore, this review did not assess the magnitude of association between individual biomarkers and recurrence risk, as most included studies did not report effect sizes; this is an important area for future research. Future scoping or systematic reviews focused on genetic and molecular markers could provide additional insight into this promising area.

## Conclusion

In conclusion, this review sheds light on the complex and multifaceted realm of breast cancer recurrence markers, while also unveiling promising paths forward for improving patient outcomes.. While challenges exist, particularly concerning marker clarity and consensus among clinicians, the integration of advanced techniques and patient-centered data holds promise for personalized risk assessment and care. By incorporating patient-reported real-world data and leveraging non-invasive clinical markers alongside traditional markers, future research endeavors can offer a more comprehensive approach to breast cancer management. Ultimately, such efforts have the potential to enhance treatment strategies, improve patient outcomes, and optimize cancer care pathways, ultimately leading to better outcomes in breast cancer recurrence.

## Data Availability

The data used in the analysis is available upon a reasonable request to the corresponding author.

## Abbreviations

BC: Breast cancer
CA: Cancer antigen
CEA: Carcinoembryogenic antigen
MD: Mammographic density
METV: Most enhancing tumor volume
SUV: Standardized uptake value
MTV: Metabolic tumor volume
HER2: Human Epidermal Growth Factor Receptor 2
HIZE: High-intensity zone emphasis
HISZE: High-intensity short-zone emphasis
ALN: Axillary lymph node; TLG—total lesion glycolysis
ADC: Apparent diffusion coefficient
ER: Estrogen receptor
TIL: Tumor infiltrating lymphocites
H&E: Hematoxylin and eosin
DCIS: Ductal carcinoma in situ
AR: Androgen receptor
BPE: Background parenchymal enhancement
